# Weighted gene co-expression network-based approach to identify key genes associated with anthracycline-induced cardiotoxicity and construction of miRNA-transcription factor-gene regulatory network

**DOI:** 10.1186/s10020-021-00399-9

**Published:** 2021-11-03

**Authors:** Guoxing Wan, Peinan Chen, Xue Sun, Xiaojun Cai, Xiongjie Yu, Xianhe Wang, Fengjun Cao

**Affiliations:** 1 Department of Oncology, Renmin Hospital, Hubei University of Medicine, 39 Chaoyang Road, Shiyan, 442000 Hubei China; 2 Department of Cardiology, the Second Affiliated Hospital of Shantou University Medical College, Shantou, 515000 Guangdong China

**Keywords:** Anthracyclines-induced cardiotoxicity, Weighted correlation network analysis, Differentially expressed analysis, Regulatory network

## Abstract

**Background:**

Cardiotoxicity is a common complication following anthracycline chemotherapy and represents one of the serious adverse reactions affecting life, which severely limits the effective use of anthracyclines in cancer therapy. Although some genes have been investigated by individual studies, the comprehensive analysis of key genes and molecular regulatory network in anthracyclines-induced cardiotoxicity (AIC) is lacking but urgently needed.

**Methods:**

The present study integrating several transcription profiling datasets aimed to identify key genes associated with AIC by weighted correlation network analysis (WGCNA) and differentially expressed analysis (DEA) and also constructed miRNA-transcription factor-gene regulatory network. A total of three transcription profiling datasets involving 47 samples comprising 41 rat heart tissues and 6 human induced pluripotent stem cell-derived cardiomyocytes (hiPSCMs) samples were enrolled.

**Results:**

The WGCNA and DEA with E-MTAB-1168 identified 14 common genes affected by doxorubicin administrated by 4 weeks or 6 weeks. Functional and signal enrichment analyses revealed that these genes were mainly enriched in the regulation of heart contraction, muscle contraction, heart process, and oxytocin signaling pathway. Ten (Ryr2, Casq1, Fcgr2b, Postn, Tceal5, Ccn2, Tnfrsf12a, Mybpc2, Ankrd23, Scn3b) of the 14 genes were verified by another gene expression profile GSE154603. Importantly, three key genes (Ryr2, Tnfrsf12a, Scn3b) were further validated in a hiPSCMs-based in-vitro model. Additionally, the miRNA-transcription factor-gene regulatory revealed several top-ranked transcription factors including Tcf12, Ctcf, Spdef, Ebf1, Sp1, Rcor1 and miRNAs including miR-124-3p, miR-195-5p, miR-146a-5p, miR-17-5p, miR-15b-5p, miR-424-5p which may be involved in the regulation of genes associated with AIC.

**Conclusions:**

Collectively, the current study suggested the important role of the key genes, oxytocin signaling pathway, and the miRNA-transcription factor-gene regulatory network in elucidating the molecular mechanism of AIC.

**Supplementary Information:**

The online version contains supplementary material available at 10.1186/s10020-021-00399-9.

## Background

Anthracycline chemotherapy maintains a prominent role in the treatment of various types of cancer, such as breast cancer, sarcoma, lymphoma and leukemia. However, cardiotoxicity, a well-known and major side effect following treatment with anthracyclines, represents a serious limitation to deliver optimal chemotherapy to cancer patients (Vejpongsa and Yeh 2014). As documented, anthracyclines-induced cardiotoxicity (AIC) is cumulative and dose-dependent, and irreversible. A devastating cardiotoxic effect of doxorubicin, an typical anthracycline compound, is principally heart failure with incidence rates of 3%, 7%, and 18% subjected to an accumulative dose of 400, 550 and 700 mg/m^2^, respectively (Shabalala et al. 2017). Since the cardiotoxicity is usually manifested tardily and undetectable for many years, it remains a lifelong threat. Even after decades of investigation and extensive efforts on identifying strategies to prevent or treat AIC, little broad consensus regarding the best approach was reached due to the incomprehension of pathogenesis. Therefore, the identification of the molecular basis of AIC is desperately needed for the discovery of novel pharmacological target and potential mechanism.

Although accumulative evidence has revealed that multifactorial mechanism may account for AIC including excessive oxidative stress, inhibition of topoisomerase 2β, accumulation of toxic metabolites, inflammation, alterations in iron (Fe^2+^) and calcium (Ca^2+^) homeostasis, mitochondriopathy, ErbB2/ERbB4 and Neuregulin-1 signaling, the exact mechanism remains unclear (Corremans et al. 2019). With the application of high-throughput microarray technology, a growing of studies were conducted in attempt to investigate potential genes and mechanisms involved in AIC. However, most studies just concentrated on the screening of differentially expressed genes and did not describe functionality of a gene within a whole system or network of interacting pathways. To describe the interconnection between genes with similar expression patterns potentially associated with a feature, these genes who may be functionally related were analyzed by using the algorithm, weighted gene co-expression network analysis (WGCNA) at the whole system level and further to delineate the relationships between different functional elements (Langfelder and Horvath 2008). WGCNA is able to construct free-scale gene co-expression networks to explore the relationships between different gene sets or between gene sets and phenotypes or traits. WGCNA has been widely applied to screen for biological processes and treatment targets as well as diagnostic biomarkers related to diseases.

In the present study, we employed for the first time the WGCNA analysis with a microarray data involving a large sample size to identify key genes associated with AIC, and we further verified the finding by another transcription profiling data. The key genes cooperating the impact of doxorubicin (DOX) on cardiomyocytes were further validated in human induced pluripotent stem cell-derived cardiomyocytes (hiPSCMs) and a miRNA-transcription factor-genes regulatory network was also constructed. The finding of the study may provide new insights into the mechanism and therapeutic targets of AIC.

## Methods

### Data collection and gene expression analysis of transcription profiling

Gene expression profile dataset E-MTAB-1168 and relevant treatment data were obtained from ArrayExpress (https://www.ebi.ac.uk/arrayexpress/). E-MTAB-1168 was performed on Affymetrix GeneChip Rat Genome 230 2.0, resulting in 89 transcription profiling data of rat heart tissues treated with saline or doxorubicin 1, 2 or 3 mg/kg by intravenous injection once per week for 2, 4 or 6 weeks (66 samples for doxorubicin and 23 samples for saline). Of dataset E-MTAB-1168, samples with a cumulative dose of 12 mg/kg were selected to construct co-expression networks and identify core genes associated with AIC. Expression profile datasets GSE154603 and GSE157282 were downloaded from Gene Expression Omnibus (GEO) database (http://www.ncbi.nlm.nih.gov/geo/). GSE154603 was performed on Illumina HiSeq 4000 (Rattus norvegicus) and comprised of 16 transcription profiling data of heart tissue of rats treated with saline (10 samples) or doxorubicin (6 samples) 3 mg/kg by intravenous injection once per week for 5 weeks. GSE157282 was performed on Illumina NovaSeq 6000 (Homo sapiens) and 6 samples of hiPSCMs treated 1 μM doxorubicin (DOX) or control were selected. Dataset GSE154603 and GSE157282 were used to verify the core genes extracted from WGCNA analysis and confirmed the key genes in the present study.

### Data preprocessing and differentially expressed genes (DEGs) analysis

Raw expression profiles data (.CEL format) were read with affy package and normalized by Robust Multiarray Average method followed by populating missing value with k-Nearest Neighbor method in R software if necessary (Irizarry et al. 2003; Troyanskaya et al. 2001). The normalized expression matrix was then annotated by the annotation file attached with the raw expression profile. The differentially expressed analysis (DEA) was performed for E-MTAB-1168 with limma R package and the differentially expressed genes (DEGs) were screened out with the criterion “FDR (false discovery rate) < 0.05 and |log2 fold change (FC)|> 1”. Moreover, the samples from the GSE154603 were divided into two groups according to the treatment (doxorubicin or control), and then the DEGs were identified using DESeq2 R package after annotating with the Rattus_norvegicus.Rnor_6.0.103.gtf. For the GSE157282 (3 doxorubicin and 3 control samples), the process was performed with the online pipeline OneStopRNAseq (Li et al. 2020) with P < 0.05 and |log2FC|> 0.96 as significant. The clustering analysis and principal component analysis (PCA) based on normalized expression matrix were performed to detect outlier samples and all differentially expressed analyses were performed following removal of the outlier samples.

### Co-expression network construction

First, the normalized expression profile data of E-MTAB-1168 was examined to evaluate whether the samples and genes were of sufficient quality. Subsequently, the genes in the top 25% of variance were selected to construct the scale-free co-expression network using the WGCNA package in R after excluding the outlier samples if any. Initially in the WCGNA, the pearson's correlation matrices and average linkage method were both performed for all pair-wise genes. Then, the correlation matrix was transformed into a weighted adjacency matrix through a power function. A gradient test was performed to analyze the scale independence and average connectivity of modules at the condition of different power values. After establishing the most appropriate power value (also called soft-thresholding parameter, β), the adjacency matrix was transformed into a topological overlap matrix (TOM), which could measure the network connectivity of a gene defined as the sum of its adjacency with all other genes for network generation, and the corresponding dissimilarity (1-TOM) was calculated. Average linkage hierarchical clustering was established in line with the TOM-based anisotropy measurement with a minimum size of 30 for the gene dendrogram to classify similar genes into one module.

### Identification of core modules correlated with treatment traits

Two approaches were utilized to identify core module correlated with DOX treatment. Gene significance (GS) was defined as the log_10_ transformation of the P value (GS = logP) on the basis of the linear regression between traits and gene expression. Additionally, module significance (MS) was calculated by the average GS of all genes in one module. Generally, the module with the absolute MS ranked first or second was considered to be the module most closely correlated with a phenotype trait. Furthermore, module eigengenes (MEs) were defined as the first principal component of a given module, which was considered a representative of the gene expression profiling. Additionally, the correlation between MEs and clinical traits (DOX treatment) were calculated to identify the relevant module. The module with the maximal correlation coefficient among all the selected modules was usually considered as the one related with DOX treatment. Combining the two methods, the module highly correlated with DOX treatment was selected ultimately for further analysis.

### Functional enrichment and protein–protein interaction (PPI) analysis of core genes and identification of hub genes

First, the common upregulated or downregulated DEGs in E-MTAB-1168 were determined by intersection of DEGs induced by 4-week and 6-week DOX treatment. Similarly, the common module genes generated by WGCNA were determined by intersection of genes in the module representative of 4-weeks and 6-weeks DOX treatment. Then, the core genes were defined as the common module genes in the common DEGs cluster. The Gene ontology (GO) including biological process (BP), molecular function (MF), and Kyoto Encyclopedia of Genes and Genomes (KEGG) enrichment analysis for the core genes were performed with the clusterProfiler (Yu et al. 2012), and the enrichment terms with a P-value < 0.05 were considered significant. The top ten terms with the least P-value were illustrated. The PPI for the core genes was also performed by using the STRING database. Additionally, the core genes were verified with the GSE154603 dataset. The shared genes between the core genes and DEGs in GSE154603 were defined as the key genes associated with the AIC and the correlation analysis between key genes were carried out with the GGally package (Barret Schloerke et al. 2021). Ultimately, the key genes cooperating the impact of DOX on cardiomyocyte were further validated by GSE157282 based on hiPSCMs.

### miRNA-transcription factor-genes regulatory network construction

The miRNAs which may target the key genes were predicted with multiMiR R package and only experimentally validated miRNAs-genes regulatory network were selected (Ru et al. 2014). The transcription factors (TF) which may regulate the key genes were predicted with ChIPBase v2.0 based on ChIP-seq data (http://rna.sysu.edu.cn/chipbase/) to construct the TF-gene network (Zhou et al. 2017). Finally, the miRNA-TF-genes regulatory network was established and visualized with Cytoscape software.

## Results

### Weighted co-expression network construction and significant modules identification

**Fig. 1:**
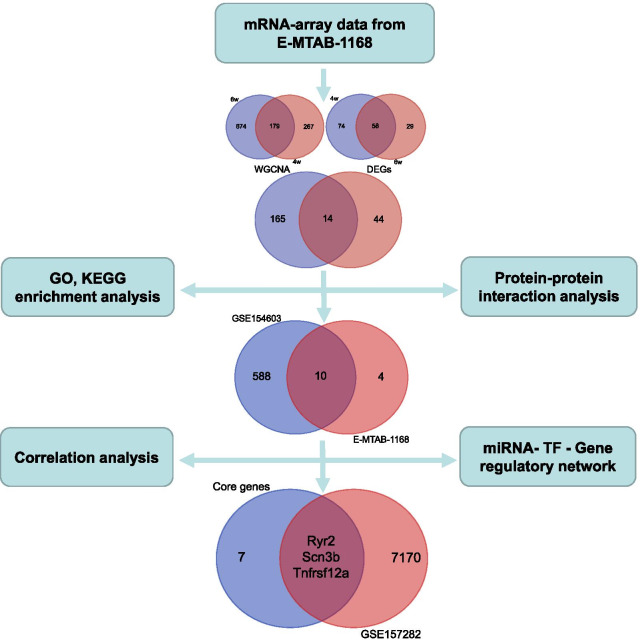
Flow diagram of the analysis procedure

**Fig. 2:**
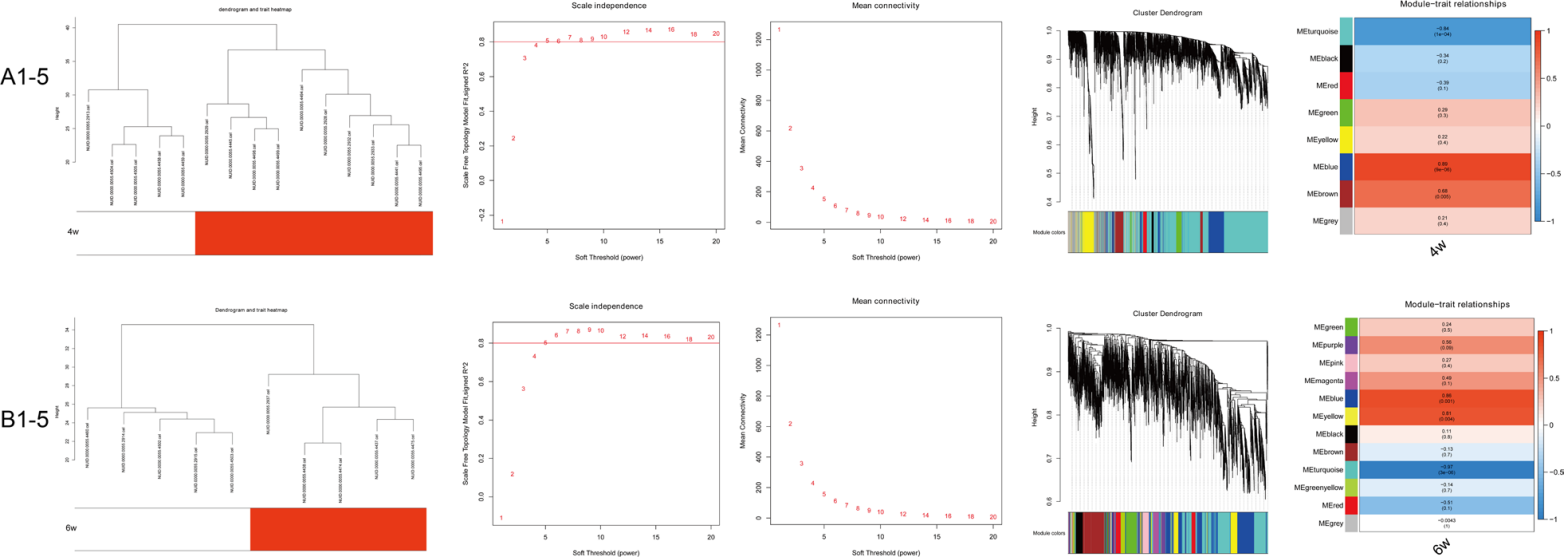
WGCNA for genes associated with AIC using E-MTAB-1168. **A1–5** for 4-week doxorubicin treatment, **B1–5** for 6-week doxorubicin treatment. **A1–5** 1–5 in order: Clustering dendrogram of rat heart tissue samples; analysis of the scale-free fit index for various soft-thresholding powers; analysis of the mean connectivity for various soft-thresholding powers; dendrogram of all differentially expressed genes clustered based on a dissimilarity measure (1-TOM); heatmap of the correlation between module eigengenes and doxorubicin treatment

**Fig. 3:**
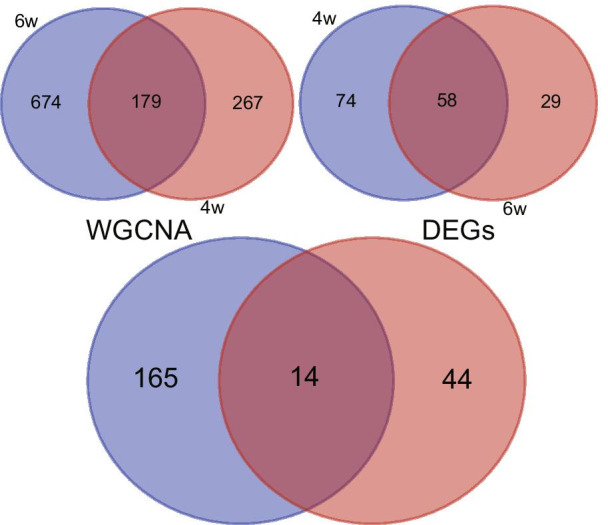
Intersection analysis WGCNA and DEA for genes modulated by 4-week and 6-week doxorubicin treatment. Figure below represents the intersection analysis WGCNA and DEA results

According to the established scheme, samples with a doxorubicin cumulative dose of 12 mg/kg administrated by 3 mg/kg per week for 4 weeks or 2 mg/kg per week for 6 weeks along with the corresponding controls in E-MTAB-1168 were selected, resulting in 15 samples (10 DOX and 5 control) for 4 weeks and 10 samples (5 DOX and 5 control) for 6 weeks after removing the outlier samples. After the quality control, the clustering analysis indicated well similarities within groups. The flow diagram of the study is shown in Fig. 1. For 4-week treatment, 3739 in the top 25% of variance out of total 14,956 genes from 15 samples were kept for the WGCNA analysis in the study. As clustered in Fig. 2A, the selected 3739 genes with similar expression patterns were grouped into modules via the average linkage hierarchical clustering and Pearson’s correlation method. An unsigned scale-free network was constructed with the most appropriate power of the soft-thresholding automatically selected by the WGCNA software, and 8 modules were excavated when satisfied the minimal 30 genes in the module. As shown in Fig. 2A, the results showed that the blue module exhibited a higher correlation with doxorubicin treatment than other modules (P = 9 × 10^–6^, R^2^ = 0.89). Therefore, the blue module involving 446 genes was identified as the clinical significant module associated with AIC for 4-week treatment. Similarly, as shown in Fig. 2B, for 6-week treatment, the WGCNA process suggested 12 modules for consideration. The blue module involving 853 genes was identified as the clinical significant module with a higher correlation with AIC (P = 0.001, R^2^ = 0.86). To assess the robustness of the genes suggested by WGCNA, only the common genes between 4-week and 6-week treatment were considered to be potentially associated with AIC, which resulted in 179 genes for further analysis (Fig. 3).

### Identification of differentially expressed genes (DEGs) and core genes

The approach of differentially expresses analysis was also applied to excavate genes associated with AIC. The expression matrices of 4-week and 6-week treatment in E-MTAB-1168 were obtained after data preprocessing abovementioned. For the 4-week treatment, 132 DEGs including 85 upregulated and 47 downregulated genes were identified with the criterion FDR < 0.05 and |log_2_FC|> 1. Similarly, for the 6-week treatment, 87 DEGs including 67 upregulated and 20 downregulated genes were identified. As a result, 58 common DEGs among 4-week and 6-week treatment were revealed by using the Venn diagram (Fig. 3). Furthermore, as previously defined, 14 core genes were identified according to the intersection analysis of 179 genes suggested by WGCNA and 58 common DEGs suggested by differentially expresses analysis (Fig. 3). The 14 core genes were considered significant for AIC.

### Functional enrichment analysis, protein–protein interaction (PPI) construction and verification of core genes

**Fig. 4:**
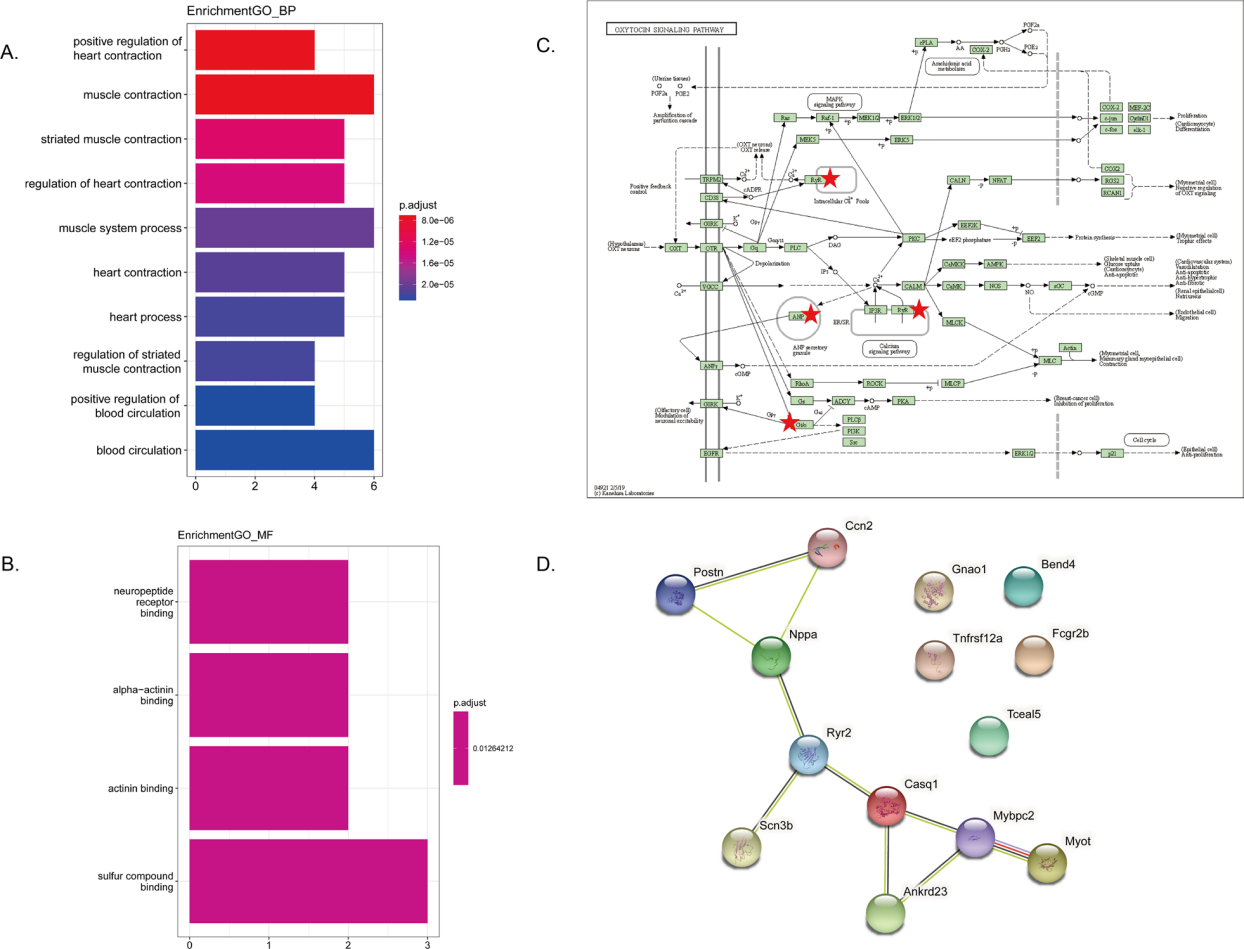
Enrichment analysis and PPI analysis. **A** Biological process in the functional enrichment analysis for the 14core genes. **B** Molecular function in the functional enrichment analysis for the 14 core genes. **C** Oxytocin signaling pathway revealed by KEGG enrichment analysis for the 14 core genes. The star represents the core genes in oxytocin signaling pathway. **D** PPI analysis using STRING database

**Fig. 5:**
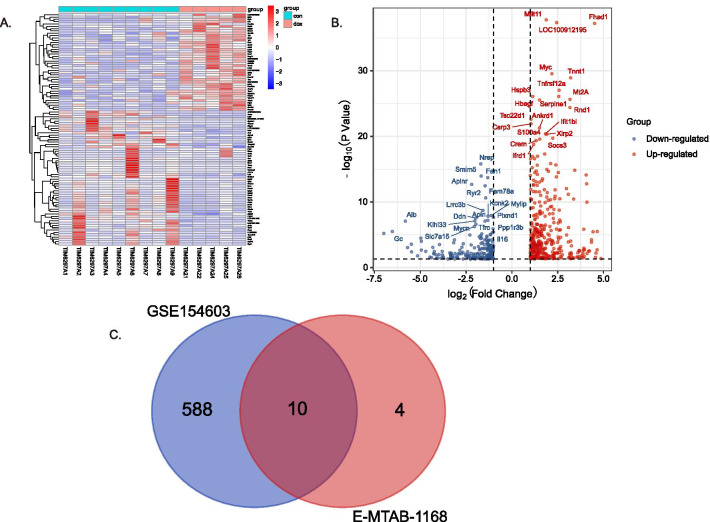
DEA to verify the 14 core genes. **A** Heatmap of the DEGs from GSE154603 dataset. **B** Volcano plot of the DEGs from GSE154603 dataset. **C** Intersection analysis between DEGs and the 14 core genes for a verification

**Fig. 6:**
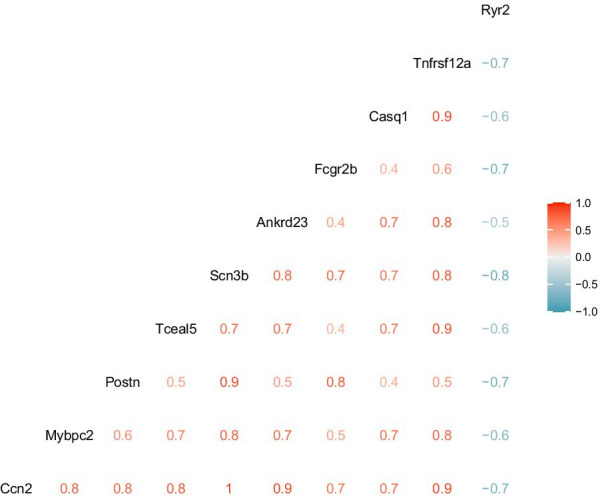
Correlation analysis for the 10 core genes with the verification dataset GSE154603

GO and KEGG enrichment analysis were performed using clusterProfiler to explore the function and pathways of the 14 core genes. As shown in Fig. 4A–C, the results for GO enrichment analysis showed that the core genes were mainly enriched in BP terms including positive regulation of heart contraction, muscle contraction, striated muscle contraction, regulation of heart contraction, muscle system process, heart contraction, heart process, regulation of striated muscle contraction, positive regulation of blood circulation, blood circulation; in MF terms including neuropeptide receptor binding, alpha-actinin binding, actinin binding and sulfur compound binding. KEGG enrichment analysis showed that the core genes were mainly enriched in oxytocin signaling pathway. Furthermore, the PPI for the core genes was also constructed and illustrated as Fig. 4D using the STRING database. To further verify the core genes, another transcription profiling data GSE154603 with 16 samples treated with doxorubicin or saline was employed. Following data preprocessing and quality assessment, 9 DOX samples and 5 control samples were enrolled for analysis after removing one DOX and one control outlier samples. Five hundred and ninty-eight DEGs including 214 downregulated and 384 upregulated genes were identified. The heatmap and volcano plots for the DEGs analysis is shown in Fig. 5. Following an intersection analysis between the 14 core genes and 598 DEGs from GSE154603, 10 core genes were verified. The gene expression changes in different datasets were listed in Table 1. In light of the interaction suggested by PPI, the correlations among the 10 verified core genes were analyzed and presented as Fig. 6.

**Table 1:** Validated DEGs in doxorubicin-induced cardiotoxicity

Gene symbol	Gene ID	E-MTAB-1168 (12 mg/kg)		GSE154603 (15 mg/kg)	Change	Gene symbol	GSE157282 (1 μM)	Change
		4 week	6 week	5 week			HiPSCM (24 h)	
Casq1	686019	1.209	1.180	1.572	Up	CASQ1	–	–
Fcgr2b	289211	1.436	1.052	1.356	Up	FCGR2B	–	–
Postn	361945	1.446	1.735	1.422	Up	POSTN	–	–
Tceal5	680282	2.452	1.611	1.449	Up	TCEAL5	–	–
Ryr2	689560	− 1.382	− 1.355	− 1.463	Down	RYR2	− 0.962	Down
Ccn2	64032	1.250	1.067	2.135	Up	CCN2	–	–
Tnfrsf12a	302965	1.202	1.047	2.577	Up	TNFRSF12A	− 2.350	Down
Mybpc2	292879	1.460	1.648	1.475	Up	MYBPC2	–	–
Ankrd23	316330	2.128	1.459	2.133	Up	ANKRD23	–	–
Scn3b	245956	1.793	1.435	2.975	Up	SCN3B	1.938	Up

*hiPSCM* human induced pluripotent stem cell (hiPS)-derived cardiomyocyte

### miRNA-TF-gene regulatory network construction and key genes identification

**Fig. 7:**
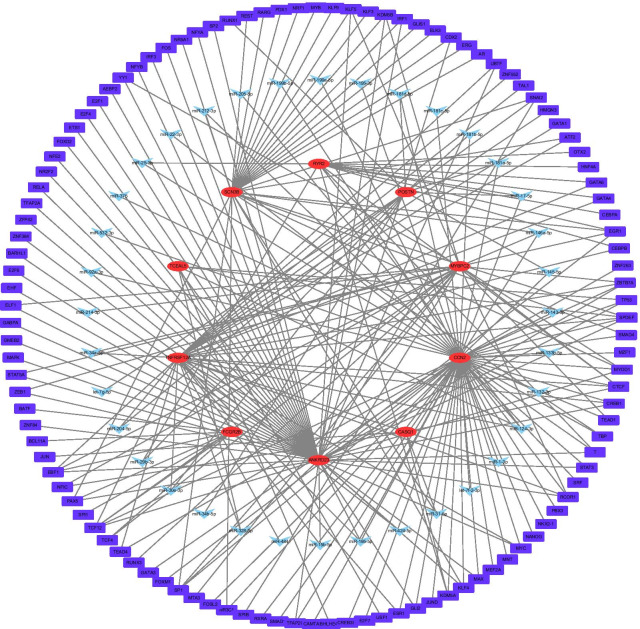
The miRNA-TF-gene regulatory network. miRNAs targeting the 10 verified core genes were predicted with multiMiR and TF targeting the 10 verified core genes were predicted with ChIPBase v2.0 online tool

Then, we explored the potential TF and miRNAs which may target the 10 core genes by using the multiMiR software and ChIPBase v2.0 online tool. The miRNAs-genes and TF-genes relationships were both experimentally validated. The regulatory network of miRNA-TF-gene was established, as shown in Fig. 7, involving 111 TFs, 36 miRNAs and 10 core genes. In this network, there were 60 miRNA-gene interaction pairs and 184 TF-gene interaction pairs. In addition, the topological properties of the regulatory network were analyzed with NetworkAnalyzer plug‑in in the Cytoscape. The top six core genes, miRNAs and TFs with high degree were listed in Table 2. To ensure the rationality of the finding applied to people, the core genes were further confirmed by the data of GSE157282 based on hiPSCMs. Six samples in GSE157282 including three treated with 1 μM doxorubicin and three treated with control were applied to perform differentially expresses analysis, resulting in 7173 DEGs. Three core genes Ryr2, Tnfrsf12a and Scn3b shared with 7173 DEGs were confirmed as key genes associated with AIC. The heatmap, volcano plots for the DEGs analysis and intersection analysis are shown in Additional file 1: Fig. S1.

**Table 2:** Topological parameters of key nodes

Gene symbol	Attribution	Average shortest pathLength	Betweenness centrality	Closeness centrality	Degree
Ccn2	Core gene	2.12179487	0.50864445	0.47129909	64
Ankrd23	Core gene	2.31410256	0.38657305	0.43213296	55
Tnfrsf12a	Core gene	2.55769231	0.17317216	0.39097744	30
Scn3b	Core gene	2.62179487	0.1790707	0.38141809	29
Mybpc2	Core gene	2.73717949	0.10746715	0.36533958	23
Ryr2	Core gene	2.81410256	0.06456454	0.35535308	14
Tcf12	TF	2.28205128	0.05201617	0.43820225	5
Ctcf	TF	2.58974359	0.03352043	0.38613861	5
Spdef	TF	2.21794872	0.04987317	0.45086705	5
Ebf1	TF	2.82051282	0.018577	0.35454545	4
Sp1	TF	2.32051282	0.02773417	0.43093923	4
Rcor1	TF	2.33333333	0.02424099	0.42857143	4
miR-124-3p	miRNA	2.75641026	0.01849095	0.3627907	4
miR-195-5p	miRNA	2.42307692	0.01558517	0.41269841	3
miR-146a-5p	miRNA	2.67948718	0.01365674	0.37320574	3
miR-17-5p	miRNA	2.92307692	0.01346221	0.34210526	3
miR-15b-5p	miRNA	2.98717949	0.00362107	0.33476395	2
miR-424-5p	miRNA	2.98717949	0.00362107	0.33476395	2

*TF* transcription factor

## Discussion

Cardiotoxicity is a very common side effect in cancer patients receiving anthracyclines chemotherapy, however, its exact mechanism remains unclear. In the present study, we, for the first time employed a systematic biology approach to explore the molecular mechanism of AIC at a systems level by integrating several genome-wide gene expression datasets and further validated the results in hiPSCMs in attempt to verify the applicability in human disease. Our study cooperating the largest sample size to date was also the first study to investigate the mechanism of AIC with multi-validation method. In the study, we constructed co-expression modules associated with AIC by the WGCNA and DEA methods, resulting in ten core genes which were verified by several different transcription profiling datasets. Of them, three key genes Ryr2, Tnfrsf12a and Scn3b were further validated in hiPSCMs. Signal pathway enrichment analysis suggested that oxytocin signaling pathway might be of importance for AIC. Moreover, a miRNA-TF-gene regulatory network was constructed for the better understanding of AIC mechanism. Most of them are rarely reported in AIC.

Dose-related cumulative and irreversible cardiotoxicity is a common and serious complication associated with the clinical use of anthracyclines. In the present study, a cumulative dose of 12–15 mg/kg was deemed appropriate to mimic the doxorubicin cardiotoxicity in the animal model (Desai et al. 2013, 2014; Gava et al. 2013; Kharin et al. 2013). Initially, microarray-based gene expressions of rats receiving a cumulative dose of 12 mg/kg doxorubicin administrated with 3 mg/kg once per week for 4 weeks or 2 mg/kg once per week for 6 weeks along with corresponding controls in the E-MTAB-1168 study were selected to identify genes associated with AIC. To minish the possible effect of different administration schemes, we applied two methods: WGCNA and DEA. We initially identified 179 genes by WGCNA between 4 and 6 weeks. Similarly, 58 DEGs were obtained by DEA. The same DEGs between 4 and 6 weeks revealed that there were some similarities in biological foundation of AIC with same cumulative dose of doxorubicin regardless of different administration schemes. However, the WGCNA method seemed to be capable of discovering more potential important genes. To enhance the reliability of genes associated with AIC, the intersection analysis between WGCNA and DEA was performed, resulting in 14 genes which were identified as the core genes. GO enrichment analysis revealed that the 14 core genes were likely to be mainly involved in the regulation of heart contraction, muscle contraction and heart process, which fitted well with concept of a myocardial injury phenotype in AIC as described previously. KEGG enrichment analysis revealed that oxytocin signaling pathway may be significant for AIC. Although few study has investigated the role of oxytocin signaling pathway in AIC, a previous study demonstrated that oxytocin may have a therapeutic effect in doxorubicin-induced cardiomyopathy in a rat in-vivo model (Taskiran et al. 2019). Moreover, oxytocin signaling pathway was found to be inhibited at various degrees in ischemia/reperfusion-induced injury and atherosclerotic cardiovascular disease, and preclinical studies have also demonstrated its cardioprotective effects (Jankowski et al. 2020; Wang et al. 2019; Xiong et al. 2020). These findings suggest that oxytocin signaling pathway may be inhibited by doxorubicin in cardiomyocyte, which is needed to be validated by further experiments. In addition, the PPI analysis was performed to analyze the correlations among the core genes. The results suggested an underlying relationship among several genes (Fig. 4D), which was supported by the correlation analysis (Fig. 6). However, the underlying mechanism remains to be elucidated.

To validate the core genes, we applied a mRNA-seq gene expression profile to perform DEA. Ten shared with the 14 core genes including nine upregulated genes (Casq1, Fcgr2b, Postn, Tceal5, Ccn2, Tnfrsf12a, Mybpc2, Ankrd23, Scn3b) and one downregulated gene (Ryr2) were identified, which were deemed significant. Some of them have been reported to be implicated in the development of AIC. For example, the expression of pro-fibrotic cytokine connective tissue growth factor Ccn2, also known as Ctgf, was increased by doxorubicin treatment, but found reduced following lovastatin protection against AIC. Consistent with our finding, previous studies have demonstrated the important role of Ryr2 in AIC(Pessah, 1992; Takaseya et al. 2004). Doxorubicin could induce calcium release of sarcoplasmic reticulum to induce AIC by directly binding to the cardiac-type ryanodine receptor to inhibit the activity of Ryr2 (Hanna et al. 2017; Saeki et al. 2002). These findings along with our results suggest that the regulation of Ryr2 expression or activity may be a potential strategy for the treatment of AIC. Although the rest novel genes including Casq1, Fcgr2b, Postn, Tnfrsf12a, Ankrd23 and Scn3b have never been reported in AIC, several of them have been demonstrated associated with cardiomyopathy, myocardial ischemia–reperfusion injury, viral myocarditis, fibrosis, cardiac remodeling, arrhythmia and cardiac hypertrophy disease, respectively (Hasdemir et al. 2010; Li et al. 2009; Ma et al. 2016; Yao et al. 2020; Zhao et al. 2018).

Furthermore, a mRNA-seq gene expression profile based on hiPSCMs was used to verify our finding for generalization in human disease. As a result, three key genes (Ryr2, Tnfrsf12a and Scn3b) were identified. As expected, the changes of Ryr2 and Scn3b expression in hiPSCMs were consistent with that in animal models. The role of Ryr2 in AIC has been discussed above. Although the role of Scn3b in AIC has not been reported ever, the increased expression of Scn3b modulated by interleukin 2 was found to be associated with arrhythmia (Zhao et al. 2016). On the contrary, Scn3b knockout exhibited abnormal sino-atrial and cardiac conduction properties in mice (Hakim et al. 2010). These findings implied the importance of stabilizing Scn3b in maintaining normal rhythm. Considering the commonplace of arrhythmia events in patients treated with doxorubicin, it is biologically plausible to attach importance to the crucial role of Scn3b in AIC. However, the changes of Tnfrsf12a expression induced by doxorubicin in hiPSCMs was contradictory to that in the heart tissues in animal models. Given the knowledge that the pathomechanism of AIC is complicated including inflammatory response, fibrosis, oxidative damage, et al. and doxorubicin usually induces proliferation of cardiac fibroblasts but apoptosis in heart, inflammatory and fibrotic factors is momentous in mediating AIC (Cappetta et al. 2017). Tnfrsf12a also known as Fn14 (fibroblast growth factor-inducible 14) or Tweakr is a member of TNF receptor superfamily which play a vital role in mediating fibrosis and ubiquitously expressed in various cells especially cardiac fibroblasts in the heart (Das et al. 2018; Lyu et al. 2018; Novoyatleva et al. 2013). Hence, there is a possibility to explain the conflict that the different expression pattern of Tnfrsf12a in heart tissues and cardiomyocytes induced by doxorubicin may be associated with different effects of doxorubicin on cardiac fibroblasts and cardiomyocytes.

Additionally, a regulatory network, containing the genes, TFs and miRNAs, was constructed for a better understanding of the core genes in the development of AIC. According to topological property analysis, several top-ranked TFs and miRNAs were identified as potential factors affecting the core genes (Table 2). In fact, some of them have been reported to play a role in AIC in previous studies. For example, previous study found that interfering with the stability of the Sp1/Stat3 transcription complex by atorvastatin protected effectively cardiomyocyte from doxorubicin toxicity by modulating survivin expression (Oh et al. 2020). Specific silencing of the top-ranked miR-146a-5p was found to increase doxorubicin-induced cardiomyocytes death by suppressing its targets (Milano et al. 2020). Whereas, upregulation of miR-17-5p induced by dexrazoxane exhibited a cardioprotective role against doxorubicin-induced apoptosis of cardiomyocyte (Yu et al. 2020). Our previous study found that miR-15b-5p was involved in doxorubicin-induced cardiotoxicity via inhibiting Bmpr1a Signal in H9c2 cardiomyocyte (Wan et al. 2019). Although the exact mechanism remains largely unknown, the regulatory network we developed may provide important clues for further experimental validation and investigation regarding combinational regulation of miRNAs and TFs on the core genes in AIC.

Several limitations should be acknowledged in the present study. First, although we can propose some target genes involved in AIC by external validation using transcriptome data of rats and human, the sample size in the discovery (E-MTAB-1168) and validation datasets (GSE154603 and GSE157282) are small and our findings warrant further validation by molecular biology experiments. Second, the animals used in the current study do not have cancers and some confounding factors such as co-morbidities and concomitant medications are not considered. However, some modifications inherent to cancer pathophysiology as well as the confounding factors potentially impact the degree of AIC. Thus, future experimental design should take these conditions into account as much as possible. Third, variability in response to doxorubicin among different species due to the discrepancy in metabolism, genome, physiology conditions widely exist, therefor the data obtained using rats should be generalized cautiously. Fourth, changes in gene expression may not necessarily be reflective of changes in protein levels, the causal role of identified genes in the pathogenesis of AIC should be further verified at protein level.

## Conclusions

In conclusion, our study identified several key genes particularly Ryr2, Tnfrsf12a and Scn3b and the oxytocin signaling pathway in association with AIC. We also constructed a regulatory network to better understand the potential mechanism of these key genes in the development of AIC. These findings provide novel insights into the pathogenesis of AIC and may aid the exploration of therapeutic strategy for AIC. However, these findings require validation from further advanced experiments.

## Supplementary Information

**Additional file 1: Fig. 1.** DEA to validate the core genes with hiPSCMs-based GSE157282 dataset. A. Heatmap of the DEGs from GSE157282 dataset. B. Volcano plot of the DEGs from GSE157282 dataset. C. Intersection analysis between DEGs from GSE157282 dataset and the 10 core genes for a validation.

## Data Availability

The data that support the findings of this study are available from ArrayExpress E-MTAB-1168, and GEO datasets GSE154603 and GSE157282.

## Abbreviations

WGCNA: Weighted correlation network analysis
AIC: Anthracyclines-induced cardiotoxicity
DEA: Differentially expressed analysis
hiPSCMs: Human induced pluripotent stem cell-derived cardiomyocytes
DOX: Doxorubicin
GEO: Gene Expression Omnibus
DEGs: Differentially expressed genes
PCA: Principal component analysis
PPI: Protein–protein interaction
GO: Gene ontology
KEGG: Kyoto Encyclopedia of Genes and Genomes
TF: Transcription factor
