# Supplementary material for: Weighted gene co-expression network-based approach to identify key genes associated with anthracycline-induced cardiotoxicity and construction of miRNA-transcription factor-gene regulatory network
Source: Mol Med. 2021 Nov 3;27:142. doi: 10.1186/s10020-021-00399-9 (PMC8567557; doi:10.1186/s10020-021-00399-9)
Supplement: Supplementary file 1 — Additional file 1: Fig. 1. DEA to validate the core genes with hiPSCMs-based GSE157282 dataset. A. Heatmap of the DEGs from GSE157282 dataset. B. Volcano plot of the DEGs from GSE157282 dataset. C. Intersection analysis between DEGs from GSE157282 dataset and the 10 core genes for a validation. [file 10020_2021_399_MOESM1_ESM.docx]

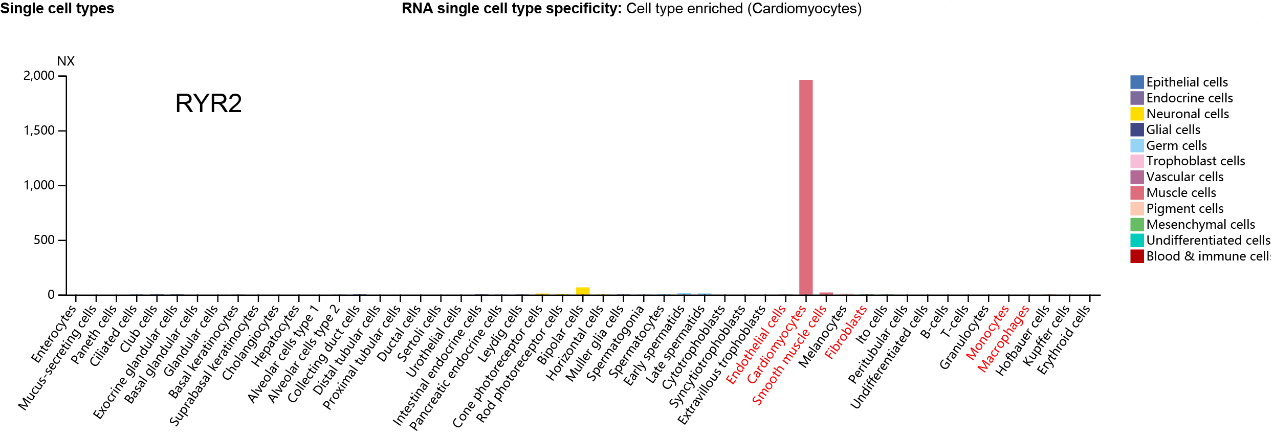


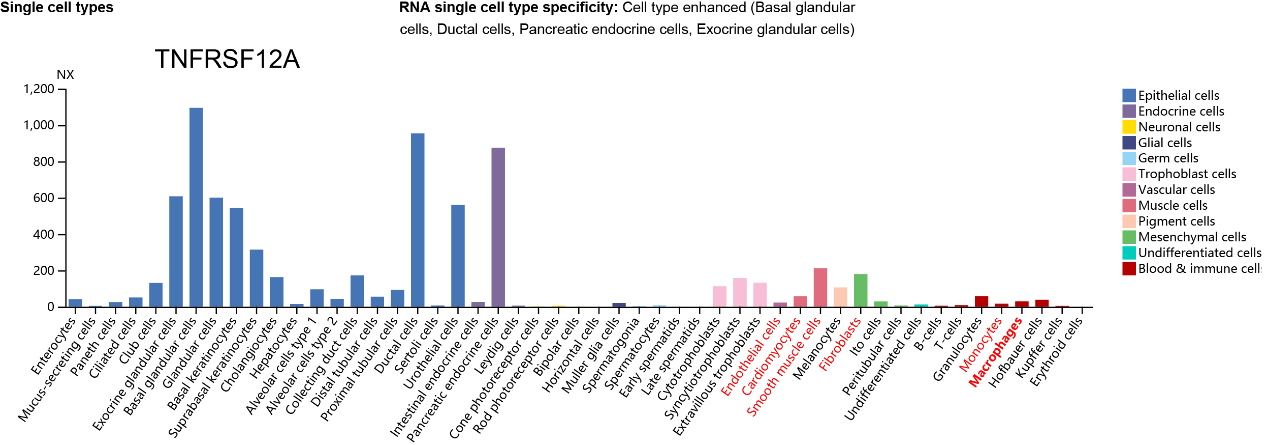


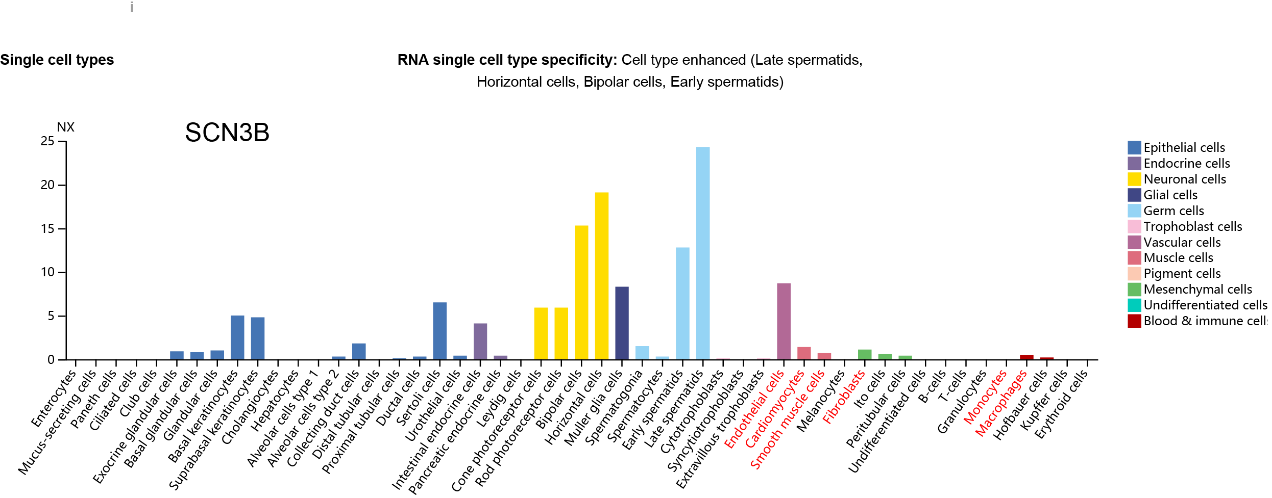


Figure legend. The mRNA expression patterns of Ryr2, Tnfrsf12a and Scn3b at single cell level were searched in the Human Protein Atlas database (https://www.proteinatlas.org/) and presented here. We highlighted several cell types enriched in heart in red.
